# Contraction of the Prepuce

**Published:** 1846-08

**Authors:** 


*Contraction of the Prepuce.*—At the Medical Soicety of London, Dr. Golding Bird alluded to a case occasionally occurring in practice, but, he believed, not mentioned in any printed work. Last Friday week, among the out-patients at Guy's, was a little child, seven or eight weeks old, lying in a state apparently comatose. The mother stated that the child had been heavy and stupid ever since its birth, and the last few days more particularly so. It fell into a lifeless state immediately after sucking from the bottle from which it was fed. The first suspicion on his mind was, that the child had taken an opiate, but this was denied by the mother, and he believed her. On opening the eyelids, the pupils were observed to act lazily, and they contracted feebly. No blow or any kind of injury had been received, If not the result of an opiate, on what did the symptoms depend? The head was not large enough to raise the suspicion of congenital hydrocephalus; nor had any symptom been presented of the acute form of that disease. On inquiring if the child passed water, the answer led to an examination of the prepuce, which was found to be elongated, and had an aperture only of the size of a pin-hole, like a puncture in the intestine. The urine was dribbling out; it was evident that the child had never completely emptied the bladder. Mr. Hilton slit up the prepuce, and all the symptoms were immediately relieved, and soon entirely removed. Dr. Bird referred to a case which he had related to the Society some years since,—and which was reported in the Lancet at the time,—of a child who fell a victim to a malformation of this kind; and after death the bladder and ureter were found like those of a man who had long suffered from stricture.

Mr. Linnecar had lately attended a gentleman with gonorrhœa, who had only a pin-like opening in the prepuce, the meatus being, however, of the natural size. This gentleman was always three-quarters of an hour in evacuating his bladder. During the efforts of micturition, the prepuce filled up like an apple.

Mr. Hilton had seen many cases similar to the one mentioned by Dr. Bird. The greatest benefit resulted from slitting up the prepuce. In this case the benefit was very remarkable, a partial paralysis of the left side, under which the little patient laboured, being quite removed in twenty-four hours.

Mr. Bransby Cooper said that it was a remark of the late Sir Astley Cooper, that it was a matter of great importance to open the prepuce in these cases of obstructed micturition; for every case of cancer which had come under the notice of that distinguished surgeon, had been associated with the condition of the prepuce alluded to. Sir Astley preferred circumcision of the prepuce to slitting it up. The fresh accumulation behind the prepuce set up inflammatory action, which, being long continued, was liable to lead to the production of cancer. —*London Lancet.*

